# A Cautionary Note on the Use of Split-YFP/BiFC in Plant Protein-Protein Interaction Studies

**DOI:** 10.3390/ijms15069628

**Published:** 2014-05-30

**Authors:** Anneke Horstman, Isabella Antonia Nougalli Tonaco, Kim Boutilier, Richard G. H. Immink

**Affiliations:** 1 Plant Research International, Droevendaalsesteeg 1, 6708 PB Wageningen, The Netherlands; E-Mails: anneke.horstman@wur.nl (A.H.); inougalli@gmail.com (I.A.N.T.); kim.boutilier@wur.nl (K.B.); 2 Physiology of Flower Bulbs, Department of Plant Physiology, Wageningen University, Droevendaalsesteeg 1, 6708 PB Wageningen, The Netherlands

**Keywords:** protein-protein interaction, split-YFP (yellow fluorescent protein), bimolecular fluorescence complementation (BiFC), protein complementation assay (PCA), *in planta*, fluorescence microscopy

## Abstract

Since its introduction in plants 10 years ago, the bimolecular fluorescence complementation (BiFC) method, or split-YFP (yellow fluorescent protein), has gained popularity within the plant biology field as a method to study protein-protein interactions. BiFC is based on the restoration of fluorescence after the two non-fluorescent halves of a fluorescent protein are brought together by a protein-protein interaction event. The major drawback of BiFC is that the fluorescent protein halves are prone to self-assembly independent of a protein-protein interaction event. To circumvent this problem, several modifications of the technique have been suggested, but these modifications have not lead to improvements in plant BiFC protocols. Therefore, it remains crucial to include appropriate internal controls. Our literature survey of recent BiFC studies in plants shows that most studies use inappropriate controls, and a qualitative rather than quantitative read-out of fluorescence. Therefore, we provide a cautionary note and beginner’s guideline for the setup of BiFC experiments, discussing each step of the protocol, including vector choice, plant expression systems, negative controls, and signal detection. In addition, we present our experience with BiFC with respect to self-assembly, peptide linkers, and incubation temperature. With this note, we aim to provide a guideline that will improve the quality of plant BiFC experiments.

## 1. Introduction

**Figure 1:**
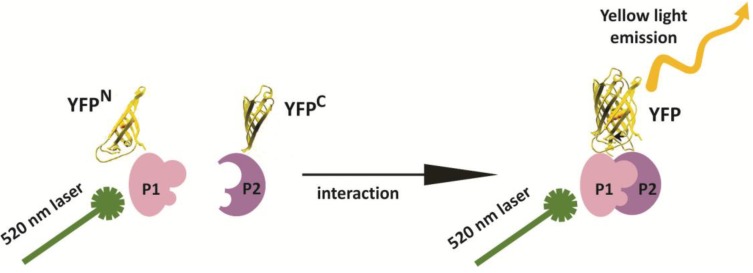
Schematic representation of the split-YFP (yellow fluorescent protein)/BiFC (bimolecular fluorescence complementation) method based on the YFP. YFP is split into two non-fluorescent “halves”, an *N*-terminal part/half of the protein (YFP^N^) and a *C*-terminal part/half of the protein (YFP^C^), which are then fused to the proteins of interest (P1 and P2). The YFP molecule is reconstituted upon interaction between P1 and P2, resulting in yellow fluorescence when the ijms are excited with the correct wavelength.

The vast majority of proteins encoded by a genome function in multi-protein complexes [1]. Identifying these protein-protein interactions can provide insight into the functions of individual proteins, as well as the biological processes they control. A large variety of high-throughput technologies have been developed in the past 20 years to identify protein-protein interactions, including a toolbox of techniques to detect or confirm putative interactions *in vivo* under physiologically relevant conditions [1,2]. The bimolecular fluorescence complementation (BiFC) assay, also referred to as “split-fluorescent protein” technology (e.g., split-YFP), is one of the most popular and frequently used methods in the plant field to study protein-protein interactions *in vivo* (reviewed in [3]). BiFC is based on the *in vivo* reconstitution of fluorescence after two non-fluorescent halves of a fluorescent protein (FP) are brought together by a protein-protein interaction event (Figure 1). As such, BiFC not only provides information on whether two proteins interact, but can also be used to determine the cellular and sub-cellular site of a protein-protein interaction event. The possibility to split a FP into two halves and to use these for the detection of interactions between ijms was first described in 2000 for the GREEN FLUORESCENT PROTEIN (GFP) [4]. Shortly thereafter, this method was used to detect *in vivo* protein-protein interactions in COS-1, NIH3T3, and HeLa cells [5], and later in plants [6,7]. The ease of implementation of the technology without the need for sophisticated equipment to detect the fluorescence signal has made BiFC a popular technology. In many cases, BiFC is the first method of choice for testing potential protein-protein interactions *in planta* and to confirm the outcomes of large-scale yeast-based or *in vitro* protein-protein interaction studies. The popularity of BiFC inspired researchers to optimize and modify the method to make it suitable for additional applications, including the development of multicolor BiFC for studying competition between interacting protein pairs or to simultaneously visualize multiple interactions in the same cell [8,9,10], and BiFC-FRET (Fluorescence Resonance Energy Transfer) for the detection of higher-order protein complex formation [11,12]. In the majority of plant studies, the split YFP-tagged proteins are overexpressed transiently or stably in isolated cells (protoplasts) or cell cultures; however, the BiFC method was recently used to study protein-protein interactions in intact plant tissues using native promoters to drive expression of the tagged proteins [13].

Despite its widespread use, the BiFC method does have a number of shortcomings for the detection and visualization of protein-protein interactions. The major drawback of the system is the ability of the two FP halves to reassemble in the absence of a *bona fide* protein-protein interaction. This so-called “self-assembly” of the FP halves can result in a high background signal, leading to a false-positive BiFC signal for a protein-protein interaction. To address this and other problems, a myriad of technical modifications have been implemented, including changing the split position in eYFP or YFP Venus from amino acid (AA) 155 to AA173 or AA210 [14], introduction of point mutations to suppress self-assembly of the two FP halves [15,16], and the use of negative controls, including point-mutated versions of the proteins under study [17]. Based on these observations, standard protocols have been developed [3,17,18] and additional optimization steps have been proposed to generate a more reliable and robust assay. Unfortunately, none of the proposed changes to improve the robustness and reliability of the method appear to be generally applicable in plants [3]. Similarly, improvements developed for a mammalian expression system did not result in a more reliable read-out in *Xenopus* [19].

We performed an inventory of the recent literature in the plant BiFC field and conclude that despite the awareness of shortcomings in the BiFC technology, the majority of researchers fail to include the correct internal controls and also incorrectly evaluate the results. Therefore, we present a guideline for BiFC use in plants, highlighting the most critical steps in the protocol and providing practical considerations for each individual step.

## 2. Results and Discussion

### 2.1. Inventory of BiFC (Bimolecular Fluorescence Complementation) Use in Plant Studies

**Figure 2:**
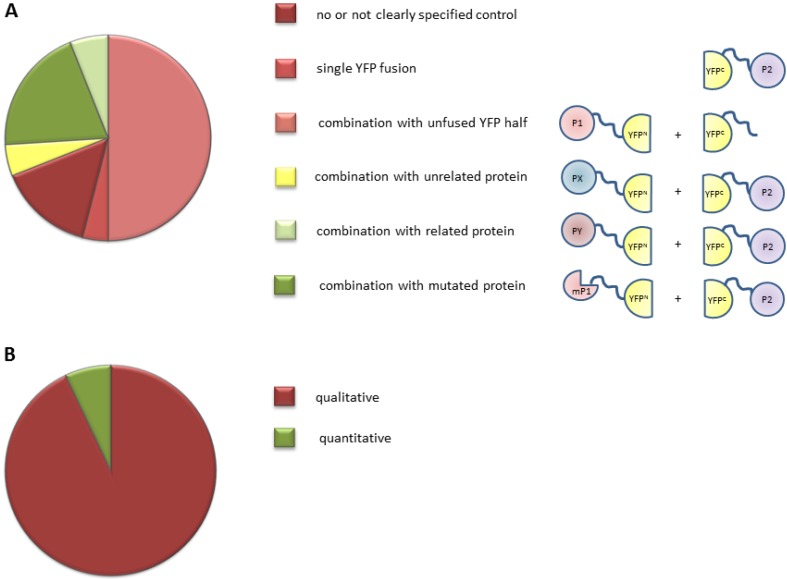
Survey of experimental set-up used in plant BiFC experiments. (**A**) Negative controls used in recent BiFC experiments. The suitability of a control is scaled using shading from dark red (the worst) to dark green (the best). The **red** classes indicate controls with a higher incidence of detecting false positives. The **green** classes represent suitable controls. The **yellow** class indicates a control of intermediate quality. Schematic representations of the controls are shown to the right. P1 and P2 represent the two proteins of interest, PX and PY indicate proteins that are related and unrelated, respectively to the protein of interest, and mP1 represents a mutant or truncated version of P1; (**B**) Percentage of BiFC experiments in which a qualitative (**red**) or quantitative (**green**) read-out of the fluorescence signal was measured.

Ten years ago, the first publications appeared showing the potential of the BiFC method for the detection and confirmation of protein-protein interactions in living plant cells [6,7]. Since then, a range of novel vectors and proposed improved protocols, mainly based on studies in mammalian cells, has been developed, with the goal to reduce the false-discovery rate and to improve the robustness of this technique. We performed a literature survey to determine which vectors, internal controls, and expression systems are used by the plant scientific community. A PubMed [20] search was performed in February 2014 using the terms “BiFC” and “plant” and the 100 most recent experimental papers were selected for analysis. From these studies, we extracted information about how the BiFC assay was performed (Table S1). Analysis of this dataset revealed that the majority of recent BiFC studies were carried out using the original vectors or using home-made vectors with the split position in the YFP molecule around amino acid 155 (AA155) [6,7]. Our analysis revealed that the newly developed vectors for the mammalian field have not been implemented in the plant field. One of the reasons might be that for plant systems, these new vectors do not solve the problem of the high false-discovery rate, as discussed below in Section 2.2.1. Remarkably and more problematic, the majority of BiFC experiments were conducted without any or with inappropriate internal controls (Figure 2A, see discussion below in Section 2.2.3). Furthermore, we noticed that in more than 90% of the studies only qualitative measurements of fluorescence signal were performed (Figure 2B, see further discussion below in Section 2.2.5).

Based on this survey, we conclude that the BiFC method is generally not executed in the proper manner. We therefore provide a guideline that can be followed for the design of an optimal BiFC experiment. This guideline is not meant to replace existing protocols (e.g., [3,17,18]), but rather, to provide additional information and notes on critical points in the method, based on published experiments and unpublished studies from our lab. This guideline will help the plant community to perform high quality BiFC studies, with an ensuing improvement in the quality of protein-protein interaction data.

### 2.2. Overview of the BiFC Method

**Figure 3:**
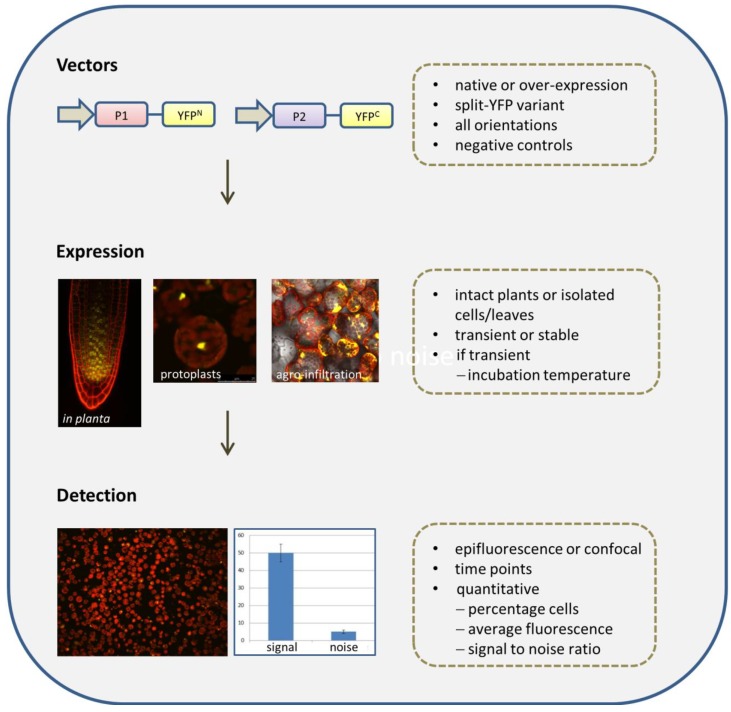
Flow diagram representing the steps and critical points in a BiFC experiment.

An overview of the BiFC method is presented in Figure 3. Prior to the start of a BiFC experiment, a number of choices have to be made, including the selection of vectors and negative and positive controls, and the expression system, each of which influences the outcome and quality of the experiment. In this section, we discuss the most important considerations for each individual step of the BiFC protocol.

#### 2.2.1. Selection of Vectors

When the BiFC method was introduced in plants in 2004, a small number of vector sets were available that all split eYFP in the loop between the seventh and eighth β-sheet (around AA155), and that expressed the fusion proteins from the strong constitutive Cauliflower Mosaic Virus 35S RNA promoter (CaMV35S) [6,7]. Unfortunately, this split position promotes irreversible self-interaction capacity of the two non-fluorescent fragments, which may result in the detection of false positive protein-protein interactions if inappropriate controls are used. A few years later, new vectors were developed that split the YFP between β-sheet nine and ten (AA173), and the resulting YFP^N^ (1–173) fragment was combined with YFP^C^ (156–239) [10]. This novel split position and combination of YFP fragments resulted in an increased signal for a positive control protein-protein interaction. However, the signal due to self-assembly also increased, which did not improve the signal (tested interaction)-to-noise (self-assembly) ratio [10]. Hence, this novel split position and combination of YFP fragments did not circumvent the problem of self-assembly and the accompanying high fluorescence signal. Aiming to minimize self-assembly, point mutations were introduced in the YFP halves, but with limited and varying success. Of all the reported mutations [3], only the amino acid change I152L in the Venus YFP protein seems to give a consistent better signal-to-noise ratio when used in animal cells [15]. However, in plant cells, this change results in a very weak fluorescence signal even with strongly interacting proteins [21].

It has also been proposed that the sequence and length of the peptide linker between the protein of interest and the YFP fragment could influence the complementation capacity of the split YFP fragments by affecting the flexibility and/or folding of the fused proteins, which in turn might be required for complex formation [22]. We tested three different vector sets varying substantially in the sequence and length of the peptide linker (Table 1) and obtained similar fluorescence complementation signals for the interacting petunia MADS (MCM1, AGAMOUS, DEFICIENS and Serum Response Factor) domain transcription factor proteins FLOWERING BINDING PROTEIN2 (FBP2) and FBP11. Our results suggest that this specific protein-protein interaction is not influenced by the characteristics of the peptide linker, but we cannot exclude that the peptide linker is of importance for proper folding and detection of interactions of other fusion proteins. Nonetheless, the observation that peptide linkers in commonly used BiFC vectors vary substantially, but still allow BiFC (see Table S1), suggests that the peptide linker sequence is not a critical factor for the success of a BiFC experiment.

In conclusion, our results and survey of the plant BiFC literature suggests that there is no evidence for the superiority of a particular BiFC vector set. Rather, it appears that reconstruction of the FP halves through protein-protein interaction depends more on the characteristics of the fused proteins than on the sequence of the YFP halves, the linker region, or the vector. Furthermore, BiFC efficiency differences have been observed between species, indicating that the cell type and the accompanying incubation conditions have a larger effect on BiFC than the vector itself. As discussed below, incorporating proper negative controls and experimental conditions seems to be of more importance for the success of a BIFC experiment.

**Table 1:** Overview of BiFC constructs with different peptide linker sequences between the coding regions of the YFP halves and the coding regions of two interacting petunia MADS (MCM1, AGAMOUS, DEFICIENS and Serum Response Factor) domain transcription factor proteins. The indicated linker lengths include amino acids (AA) encoded by parts of the multiple cloning site. Vector combinations were tested upon transient transfection of petunia protoplasts. All expression cassettes were embedded in a *pUC* vector backbone. Peptide linker sequences: Gateway-based cloning linker sequence [23]; RSIAT/KQKVMNH [5]; myc and HA tag [7].

BiFC Vector Set	Promoter	Protein of Interest	Peptide Linker	Length (AA)	YFP Part	Terminator
1 [24]	CaMV35S	FBP2	Gateway	17	YFP^N^	NOS
		FBP11	Gateway	18	YFP^C^	
2		FBP2	RSIAT	15	YFP^N^	
		FBP11	KQKVMNH	17	YFP^C^	
3 [7]		FBP2	Myc-c tag	26	YFP^N^	
		FBP11	HA-tag	25	YFP^C^	

#### 2.2.2. Fusion Orientations

**Figure 4:**
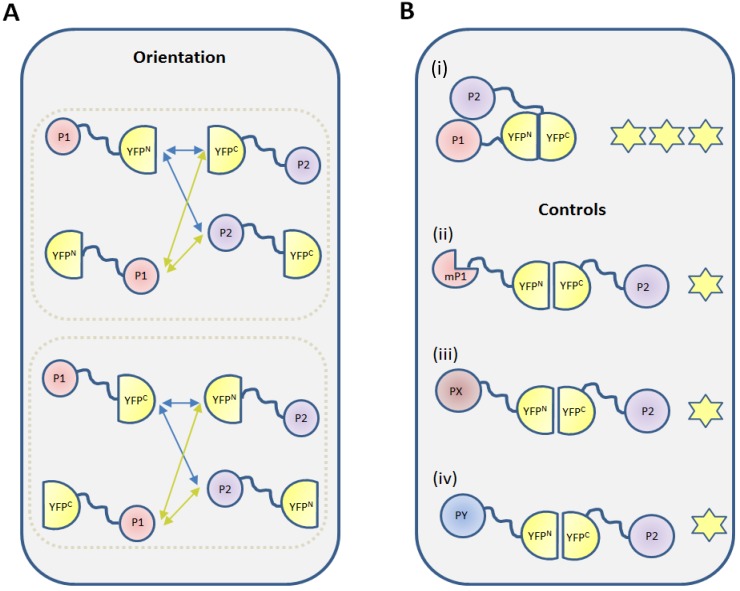
Two key elements of a BiFC experiment: fusion orientation and controls. (**A**) Each of the two YFP halves (YFP^N^ and YFP^C^) can be fused at either its *N*- or *C*-terminus, with the protein of interest. Likewise, the protein of interest can also be fused to the YFP half via its *N*- or *C*-terminus. This creates four possible YFP-protein combinations for each protein of interest and eight combinations that should be tested for each interaction pair; (**B**) In addition to the protein-protein interaction to be tested, (**i**) appropriate negative controls should be incorporated in a BiFC experiment; These negative controls include substitution for one of the protein of interest by (**ii**) a mutated protein (mP1); (**iii**) a related protein (PX) that does not interact; and (**iv**) an unrelated protein (PY) with the same subcellular localization. The stars indicate YFP fluorescence due to self-assembly (**one star**) and expression due to a *bona fide* interaction between the test proteins (**three stars**).

One factor that influences the ability to detect protein-protein interactions in BiFC assays is the effect of the YFP fusion on the protein of interest. Protein-protein interactions are mediated by specific protein domains and fusing other (fluorescent) proteins to a protein of interest can interfere with the interaction capacity of these domains by steric hindrance or due to mis-folding [25]. In addition, the three dimensional structure of a protein complex can also inhibit the reconstitution of the FP by spatial restrictions. Bracha-Drori *et al.* [6] showed that the fusion orientation can affect the amount of BiFC signal. Therefore, to exclude false negative combinations, it is recommended to generate and test all of the eight combinations of constructs in which the *N*- and *C*-terminal fragments of the FP are fused to the *N*- and *C*-terminus of the proteins of interest (Figure 4A). The functionality of these fusion proteins can also be tested by genetic complementation, provided a mutant phenotype is available for the protein of interest. We believe that a single positive combination can provide sufficient proof of protein-protein interaction, as long as suitable negative controls are included and a correct experimental set-up is followed.

#### 2.2.3. Negative Controls

The major disadvantage of BiFC as a method to detect protein-protein interactions is the signal that results from aspecific and irreversible interaction of the *N*- and *C*-terminal parts of the FP in the absence of interaction between the fused proteins of interest. For this reason, choosing a proper negative control is a critical step in the design a BiFC experiment. The ideal negative control in a BiFC experiment is a translational fusion between one half of the FP and a truncated or mutated version of the protein of interest that is unable to bind to its interaction partner (Figure 4B; e.g., [26,27]). Development of this type of negative control implies that interaction domains or amino acids have been identified using an *in vitro* approach, such as yeast two-hybrid screening, using bioinformatics predictions or genetic complementation, and that the changes to the protein do not negatively affect its stability or folding. In lieu of this, a fusion with a protein that is related to the protein of interest, but that does not interact would be a good alternative (Figure 4B; e.g., [28,29]). If a non-interacting protein family member is not known or available, one could use an unrelated, non-interacting protein with the same cellular localisation (Figure 4B). We noticed that in a few cases in the literature, separate transfection with a plasmid comprising one protein of interest fused to YFP^N^ or YFP^C^ was used as a control (Table S1). However, this control does not report the aspecific interactions that might occur between split-YFP fragments, because only one of the two YFP fragments is expressed. Empty vector controls comprising either half of the split YFP molecule, but lacking the protein of interest, were used most commonly in combination with the expression of a protein of interest fused to the complementary YFP fragment. Although it has been suggested that the expression levels of these unfused non-fluorescent fragments is higher than the expression of fusion proteins [3], which would provide a conservative background level estimation, the subcellular localization of these split-YFP fragments might differ from that of the fusion between the split FP and protein of interest, thereby abolishing any potential for aspecific interaction and subsequent underestimation of the background. Note that the FP halves of negative controls should theoretically self-assemble, but may not do so due to interference by the fused test protein. It is therefore important to always use negative control that consistently exhibits a detectable fluorescence signal to obtain a conservative estimate of the signal-to-noise ratio.

**Figure 5:**
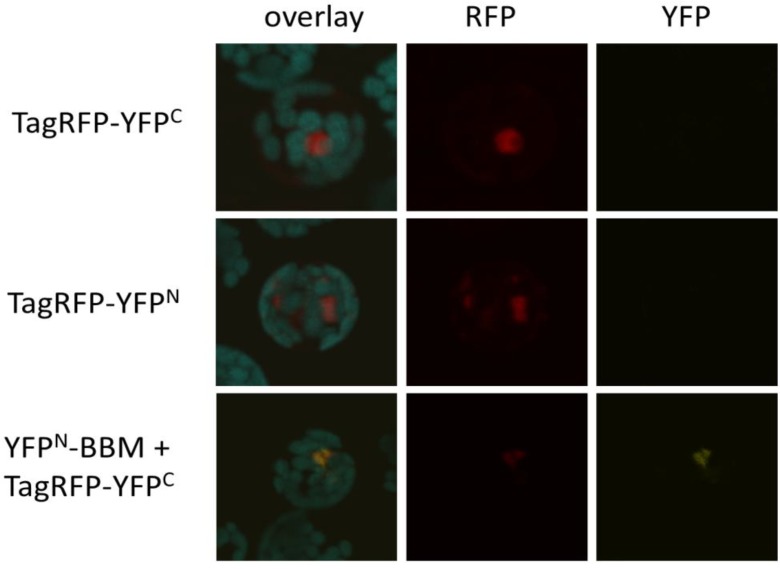
Self-assembly of YFP fragments. Confocal images of Arabidopsis protoplasts eight hours after transfection with either single TagRFP-YFP^C^ or -YFP^N^ plasmids, or with TagRFP-YFP^C^ and YFP^N^-BBM plasmid combinations. The first column shows an overlay image of the background autofluorescence signal (**blue**), the TagRFP signal (**red**) and the YFP signal (**yellow**). The second and third columns show only the TagRFP and YFP signals, respectively. Co-transfection of plasmids containing YFP^N^-BBM and TagRFP-YFP^C^ fragments results in a weak YFP signal, showing the re-constitution of the YFP molecule in the absence of a protein-protein interaction (self-assembly), and indicating the need for suitable negative controls and a quantitative read-out of the fluorescent signal in BiFC experiments.

To determine the background fluorescence levels caused by self-assembly of AA155-based BiFC vectors [24], we generated fusion constructs between TagRED FLUORESCENT PROTEIN (TagRFP) and either the *N*- or the *C*-terminal YFP fragments (TagRFP-YFP^N^ and TagRFP-YFP^C^). TagRFP is a monomeric fluorescent protein [30] and its use enabled us to confirm the expression of the individual fusion proteins. TagRFP expression was observed as early as eight hours after co-transfection of Arabidopsis protoplasts with single TagRFP fusion constructs or TagRFP-YFP^C^ and YFP^N^-BBM, a fusion of YFP^N^ with the transcription factor BABY BOOM (BBM; [31]). We noticed that in the double transfection, TagRFP fluorescence always coincided with weak YFP fluorescence. This fluorescence was not caused by bleed-through of TagRFP into the YFP channel, as no YFP signal was observed upon single transfections with TagRFP-YFP^C^ or TagRFP-YFP^N^ constructs alone (Figure 5). Since there is no indication that BBM and TagRFP proteins interact, this YFP signal likely reflects the signal from YFP self-assembly. Although the YFP signal is weak, this experiment shows that BiFC results should be interpreted with caution. BiFC experiments cannot be performed without a proper negative control: transfection with a single plasmid is not sufficient. Additionally, a quantitative read-out should be used to distinguish between a fluorescence complementation by a true protein-protein interaction and signal due to self-assembly of the two FP halves, which can be scored based on the inclusion of a suitable negative control.

#### 2.2.4. The BiFC Assay: Expression Systems

BiFC experiments in plants are almost exclusively carried out using transient expression systems, usually *Agrobacterium* infiltration of tobacco leaf cells or polyethylene glycol (PEG)-mediated transfection of leaf protoplasts. Several protocols have been published for both methods (e.g., [17,32,33,34]), and neither is considered superior. Alternatively, the BiFC assay can be performed in whole plants (*in situ*) using stable transformants in which the promoters of the protein of interest are used to drive expression of the YFP-fusion [13]. Studying protein-protein interactions in their native context ensures that any additional proteins that are required for a protein-protein interaction will be co-expressed in the correct tissue context, and that the quantitative relationship between the target protein and endogenous partner proteins is maintained. This approach can give insight about when and where a specific protein-protein interaction first occurs, however, the irreversibility of the re-assembly of the YFP halves excludes the possibility to study protein-protein interaction dynamics. Since the genomic integration site of a transgene can affect its expression, it is important to determine whether the expression level and tissue specificity/localization of the fusion protein corresponds to that of the endogenous gene, and that expression of the transgene does not confer any mutant phenotypes. Testing of multiple independent transgenic lines is therefore required. BiFC analyses *in planta* using native protein expression levels in the native cellular environment is the most elegant approach, but these type of studies are still in their infancy and need to be more thoroughly analyzed to determine their robustness and reliability.

Another factor that needs to be considered in relation to the expression system is the culture temperature of the protoplasts or plant tissues. While performing BiFC experiments by transient transfection of petunia protoplasts, we noticed a strong negative effect of the culture temperature on fluorescence complementation. Petunia protoplasts are routinely cultured at 28 °C [35,36]. A BiFC signal was not observed for the FBP2-FBP11 protein combination at this temperature, but at least 30% of the protoplasts showed a fluorescence signal when the protoplasts were incubated at 23 °C. This difference is not due to misfolding of the petunia MADS domain proteins at 28 °C, because strong fluorescence signals were obtained in protoplasts cultured at 28 °C when the FBP2 and FBP11 proteins were tagged with a complete YFP fluorophore [36,37]. The high-temperature sensitivity therefore seems to be specifically associated with the BiFC method. In support of this, a four-hour pre-incubation of mammalian cells at lower temperatures prior to BiFC imaging significantly increases the fluorescence signal [38].

Regardless of the expression method used, it is important to determine whether the control proteins and the proteins of interest are expressed. This not only provides information about the level of protein expression, which can greatly influence the results, but also indicates if the fusion between the protein of interest-split YFP is intact.

#### 2.2.5. Detection Methods

Fluorescence complementation in a BiFC experiment is usually detected using an epifluorescence microscope or a confocal laser scanning microscope (CLSM) (Supplementary Table S1). The qualitative analysis of BiFC experiments is problematic because of the self-assembly capacity of the two FP halves. Consequently, simply showing images of fluorescent cells from the protein combination of interest and non-fluorescent cells from a control transfection is insufficient proof of a protein-protein interaction. Rather, a quantitative comparison should be made between the signal obtained with the protein combination under study and the signal obtained with a proper negative control combination.

When using transient overexpression, it is important to realize that the irreversible nature of FP complementation leads to accumulation of the fluorescence signal in time, both for the tested interaction and the control experiment. The complementation of the FP by a protein-protein interaction and accumulation of fluorescence signal will proceed faster than the self-assembly in the control experiment due to the higher binding affinity of the interacting fusion proteins. Therefore, it is advisable to measure fluorescence signals at different time points after transfection, as saturation of fluorescence signals influences the signal-to-noise ratio. Unfortunately, the vast majority of BiFC experiments are performed in a qualitative manner (Figure 2; Table S1). One method for BiFC quantification is to determine the percentage of positive cells for both the controls and the tested protein-protein interaction. Alternatively, the signal (tested interaction)-to-noise (self-assembly signal) ratio can be determined by measuring fluorescence intensities. Signal intensities can be determined from fluorescence images, but this approach is time-consuming, as it requires measurement of many cells on a one-by-one basis to determine average signal intensity. A faster way to analyze average fluorescence intensity in a population of cells is by fluorometry or by flow cytometry [39]. Because the amount of expressed fusion proteins greatly influences the results, it is important to determine the expression levels of the different fusions within the population of cells by Western blotting. Subsequently, the BiFC signal intensity of the cell population can be normalized against the amount of fusion protein.

## 3. Experimental Section

### 3.1. Test of Different Peptide Linker Sequences

The effect of using different peptide linker sequences between the halves of YFP and the protein of interest was examined in petunia protoplasts. The interaction between the petunia MADS domain proteins FBP2 and FBP11 [36] was used as positive control. All vectors used for this experiment were based on the *pUC* vector backbone and are described in Table 1. Isolation and transfection of petunia W115 leaf protoplasts was performed as described previously [35,36]. After transfection, protoplasts were incubated overnight in the dark at 28 °C (according to the original protocol) or at 23 °C.

### 3.2. Self-Assembly Capacity of YFP (Yellow Fluorescent Protein) Halves

*BBM* [31] and *TagRFP* [30] cDNA entry clones (pDONR207) were used to generate the *YFP^N^-BBM*, *TagRFP-YFP^N^*, *TagRFP-YFP^C^* plasmids for Arabidopsis protoplast transfection. The plasmids were cloned using recombination into Gateway-compatible BiFC vectors [24]. Arabidopsis protoplast isolation followed the procedures described in [40], except that leaves of three to four week-old Col-0 seedlings were used. Protoplast transfections were carried out as described in [41], but with a transfection time of 10 min. Fluorescence was viewed 8 h after transfection by CLSM.

## 4. Conclusions

Split-YFP/BiFC is a widely used method for the detection and confirmation of protein-protein interactions in living plant cells. Nevertheless, the usefulness of this technology is overshadowed by self-assembly of the two halves of the FP, which results in the detection of fluorescent signal regardless of an interaction between the proteins of interest. Consequently, the introduction of control experiments is essential to obtain evidence for a potential protein-protein interaction event. However, a literature survey revealed that proper controls are missing in more than half of all analyzed studies. As discussed in Section 2.2.3, a negative control should be included for each tested protein-protein interaction, and the fluorescent signals should be measured quantitatively. Currently, images are presented of plant cells or tissues with a fluorescent signal, without providing a thorough quantification of the signal-to-noise ratio between the fluorescence signals from the proteins of interest and the negative controls. It is important to realize that the goal of a BiFC experiment is to obtain strong support of a protein-protein interaction and not just to obtain an image of a fluorescent cell.

The lack of reliability and robustness of the split-YFP/BiFC technology due to self-assembly of the FP halves was recognized shortly after the introduction of the method, and a plethora of modifications were suggested to overcome this problem, as discussed in Section 2.2.1. Various improvements have been suggested based on splitting the FP at different positions and the introduction of point mutations in the FP sequence, but these improvements only appeared to overcome the self-assembly problem for the tested protein combinations, or only under specific conditions. Therefore, we conclude that none of the currently used BiFC vectors is superior and that all can be used as long as the right controls are included and quantitative measurements are applied. Alternatively, methods based on complementation of other types of proteins, such as split-ubiquitin and split-luciferase (for review on these techniques, see [42]) can also be used. Self-assembly of protein halves is not an issue for these proteins; however, one drawback of using ubiquitin or luciferase is that due to the nature of the read-out in these systems, no information can be extracted about the subcellular position of a protein-protein interaction event. In this respect, FRET (Fluorescence Resonance Energy Transfer)-based methods are more informative than protein complementation assays, because information is obtained on both the localisation pattern of the individual proteins and of the protein-protein interaction [36,41], but these methods require sophisticated microspectroscopy equipment.

In conclusion, a low-tech, robust, and fully reliable system for the detection of protein-protein interactions in plant cells or tissues does not exist. Nonetheless, when implemented with caution split-YFP/BiFC remains a valuable tool for studying protein-protein interactions.

## Supplementary Files

Supplementary File 1 Supplementary Table (XLSX, 23 KB)
